# Experiencing maternity care: the care received and perceptions of women from different ethnic groups

**DOI:** 10.1186/1471-2393-13-196

**Published:** 2013-10-22

**Authors:** Jane Henderson, Haiyan Gao, Maggie Redshaw

**Affiliations:** 1 Policy Research Unit in Maternal Health and Care, National Perinatal Epidemiology Unit, University of Oxford, Old Road, Oxford OX3 7LF, UK

**Keywords:** Ethnic groups, Maternity care, Pregnancy, Health care surveys, England

## Abstract

**Background:**

According to the Office for National Statistics, approximately a quarter of women giving birth in England and Wales are from minority ethnic groups. Previous work has indicated that these women have poorer pregnancy outcomes than White women and poorer experience of maternity care, sometimes encountering stereotyping and racism. The aims of this study were to examine service use and perceptions of care in ethnic minority women from different groups compared to White women.

**Methods:**

Secondary analysis of data from a survey of women in 2010 was undertaken. The questionnaire asked about women’s experience of care during pregnancy, labour and birth, and the postnatal period, as well as demographic factors. Ethnicity was grouped into eight categories: White, Mixed, Indian, Pakistani, Bangladeshi, Black Caribbean, Black African, and Other ethnicity.

**Results:**

A total of 24,319 women completed the survey. Compared to White women, women from minority ethnic groups were more likely to be younger, multiparous and without a partner. They tended to access antenatal care later in pregnancy, have fewer antenatal checks, fewer ultrasound scans and less screening. They were less likely to receive pain relief in labour and, Black African women in particular, were more likely to deliver by emergency caesarean section. Postnatally, women from minority ethnic groups had longer lengths of hospital stay and were more likely to breastfeed but they had fewer home visits from midwives. Throughout their maternity care, women from minority ethnic groups were less likely to feel spoken to so they could understand, to be treated with kindness, to be sufficiently involved in decisions and to have confidence and trust in the staff.

**Conclusion:**

Women in all minority ethnic groups had a poorer experience of maternity services than White women. That this was still the case following publication of a number of national policy documents and local initiatives is a cause for concern.

## Background

In 2010, 25.1% of women giving birth in England and Wales were born outside the UK [1]. According to the 2011 Census, 86% of the population of England and Wales self-identified as White (80.0% White British), 2.2% Mixed, 7.5% Asian or Asian British (2.5% Indian, 2.0% Pakistani), 3.3% Black African, Black Caribbean or Black British, and 1% Other ethnicity [2]. This represents a significant increase since 2001 in minority ethnic groups, especially non-British White groups, Black African, Indian and Pakistani groups.

Ethnic group has been defined as “a collectivity within a larger population having real or putative common ancestry, memories of a shared past, and a cultural focus upon one or more symbolic elements which define the group’s identity…” [3]. An ethnic group is characterised by race, skin colour, national or regional origins, religion and language. It evolves over time in response to social and political attitudes and is self-defined [3].

Studies in Europe and North America have indicated that, even in a developed country context, women from minority ethnic groups have poorer pregnancy outcomes than White women. The last two UK triennial enquiries into maternal deaths found that women from minority ethnic groups were at significantly greater risk [4,5]. In particular Black African and, to a lesser extent, Black Caribbean women had significantly higher mortality rates than White women, thought to be due to later engagement with maternity services [5], though it was emphasised that some Black African women may have been newly arrived refugees or asylum seekers with a less than optimal history and circumstances. Similarly, in an analysis of severe morbidity over the period 2005–2006, after adjustment for age and socioeconomic status (SES), Black African and Black Caribbean women had twice the incidence of severe morbidity compared to White women [6].

A study based on UK birth registrations (and thus country of birth rather than ethnicity) found that low birthweight (<2500 g) was most common in babies of women born in South Asia and that women born in the Caribbean and West Africa had a higher incidence of very low birthweight babies (<1500 g) [7]. Linkage to infant death records for the years 1983 to 2001 showed that infant death rates were highest in babies of Pakistani mothers, and also high in babies of Caribbean and West African women, a finding confirmed in a more recent study of infant mortality [8]. Ethnic minority groups are more likely to live in areas of deprivation, however, analyses have shown that socioeconomic status explains little or none of the mortality differentials between ethnic groups [8,9] although it does explain some of the variation in birthweight [10].

In a study comparing birthweight of first and second generation Asians in the UK, mean birthweight after adjustment was higher in babies of second generation women [11]. This was thought to be due to improved nutrition, education, community integration, better command of English, cultural and religious beliefs and practices, and socioeconomic factors including jobs and housing [11]. Health campaigns in the 1980s, such as the Asian Women and Babies Campaign and the Hackney Health Advocacy Programme, which encouraged the use of advocates and link workers to facilitate uptake of services, led to reduced rates of labour induction and caesarean section, and increased mean birthweight [12].

One likely pathway linking ethnicity and poor perinatal outcome is antenatal care [5]. A range of studies have shown that women from minority ethnic groups, especially Black and Asian women, attend later in pregnancy and have fewer antenatal checks than White women [13-16]. They also have fewer pregnancy ultrasound scans, are less likely to attend antenatal education classes, have more hospital admissions in pregnancy, and less choice regarding place of birth. Barriers to women attending for antenatal care include language, a shortage of interpreters, advocates and link workers, and cultural attitudes towards male health care professionals [17]. Women born outside the UK, even if English speaking, may have a poor understanding of how the NHS works, and have difficulty understanding healthcare jargon [12]. Informed choice is not easily available to these women unless they have interpreters or advocates. Stereotyping and racism are also evident in some staff attitudes [13].

A population based survey in 2006 found that women from minority ethnic groups worried more about labour and delivery than White women did. In particular, although embarrassment was not a major concern, it featured more often as a significant issue for ethnic minority women than for White women [18].

The importance of training of health care professionals in cultural sensitivity and the use of interpreters, advocates and link workers has long been emphasised in government reports [5,19]. The Commission for Racial Equality (now the Equality and Human Rights Commission) published a code of practice for maternity units aimed at eliminating racial discrimination and increasing equality of opportunity. However, initiatives tended to be geographically scattered, targeted only at numerically significant minority populations and time limited [12]. More recent reports have again highlighted these problems [20,21].

There is evidence of a perception amongst some staff that if minority ethnic group women behaved more like White women, the health disadvantages of these groups would disappear [22]. Midwives stereotypically view Asian women as needing less support, being generally well supported by their families, as having a lower pain threshold in labour [16,23], that they make a fuss, are non-compliant and too demanding [16,24]. However, women of different backgrounds may respond differently to pain, for example, Pakistani women have been reported to have more open and emotional reactions than White women [25]. In a Norwegian study of 67 Pakistani women born in Punjab, there were no differences in length of labour or mode of delivery compared to 70 Norwegian born women, but there were significant differences in their use of analgesia. Getting appropriate pain relief depends on effective communication and empathy, and methods of pain relief such as epidural and spinal analgesia require information-giving, opportunities for discussion, and cooperation. No Pakistani women, only half of whom spoke Norwegian, had an epidural, even those with long labours. After adjustment for potential confounders, the only factor significantly associated with receiving pain relief was mother’s country of birth [25]. Inadequate pain relief, less confidence and trust in staff, and being left alone and worried in labour or shortly after the birth were also reported more by Asian and Black women in a UK survey [15]. However, in using simpler groupings of 'Asian’ and 'Black’ women, that study did not separate the views and experiences of women of Bangladeshi, Indian and Pakistani origin or those of Black African women and Black Caribbean women, all of whom represent rather different ethnic groups with different histories and patterns of migration to the UK.

Women of all ethnicities born outside the UK may have particular difficulty with the transition to motherhood. The effects of being away from family and isolated in a foreign country, can lead to considerable unhappiness. Cultural differences may result in different expectations: whereas women in Northern Europe are encouraged to be up and active almost immediately following a normal birth, women from other groups may view the early postnatal period as a time for rest and seclusion, anticipating support from staff, friends and family and recognition of their changed status [26,27]. The attitudes of health workers to different practices at this time may range from being responsive to being insensitive or derisive [28].

The aims of this study were to examine use of services and perceptions of maternity care among women who had recently given birth and who self-identified as coming from seven specific ethnic groups compared to women self-identifying as White. Lumping women from Black and Minority Ethnic groups together is recognised as of limited value in trying to understand differences in care associated with ethnicity, as is groupings like 'Black’ or 'Asian’. Thus, the opportunity to break down the groupings further was taken in order to better understand differences in care and perceptions of that care.

## Methods

Structured questionnaires and an information leaflet in 20 different languages were sent to more than 50,000 women aged 16 years and over living in England in 2010, excluding women whose baby had died. The questionnaire was coordinated by the Picker Institute on behalf of the Care Quality Commission and sent from 144 NHS trusts to investigate women’s experience of maternity care. Completion and return of the questionnaire was taken as consent. There was an option for women to complete the questionnaire by phone with an interpreter but this was rarely used. Women were asked about access, information, communication and choice regarding antenatal care, delivery mode and neonatal outcomes. Information regarding demographic characteristics, including age, partner status and parity were also collected. No data were collected on socioeconomic status as the focus was on patient experience [29].

Based on the UK census question, respondents were asked to identify which group they belonged to from a list of 16 categories. In the analysis, ethnicity was grouped as: White (British, Irish and any other White background), Mixed (White and Black Caribbean, White and Black African, White and Asian, Other Mixed background), Indian, Pakistani, Bangladeshi, Black Caribbean, Black African or Other ethnic group (see Table 1). The groupings used were considered appropriate bearing in mind the numbers of respondents in each ethnic minority category.

**Table 1:** Ethnic groups listed in questionnaire, and groupings (in bold) used for analysis

	**N**	**%**
**White**	**20633**	**80.9**
British	18617	73.0
Irish	102	0.4
Any other white background	1914	7.5
**Mixed**	**319**	**1.2**
White and Black Caribbean	80	0.3
White and Black African	66	0.3
White and Asian	61	0.2
Any other mixed background	112	0.4
Asian or Asian British	1714	6.7
**Indian**	**584**	**2.3**
**Pakistani**	**596**	**2.3**
**Bangladeshi**	**163**	**0.6**
Any other Asian background*	371	1.5
Black or Black British	966	3.8
**Caribbean**	**162**	**0.6**
**African**	**659**	**2.6**
Any other black background*	145	0.6
**Chinese or other ethnic group**	**687**	**2.7**
Chinese	142	0.6
Any other ethnic group	545	2.1
Not known	1169	4.6
Total	24319	100

*Any other Asian background and any other black background included with any other ethnic group.

Outcome measures relating to women’s experience were based on responses to a series of questions about antenatal care, labour and birth and postnatal care, and the overall experience of the different phases of care as rated on a five point scale ranging from “excellent” to “poor”. The study, which complied with the Helsinki Declaration, involved secondary data analysis. The original survey evaluating maternity services in England was passed by the North West 5 Multi-Centre Research Ethics Committee (07/MRE8/1).

### Statistical analysis

The associations between women’s experience and ethnic group were investigated by logistic regression models. Multinomial logistic regression was used when the dependent variable (women’s experience) had more than two categories. Odds ratios and 95% confidence intervals were calculated by comparing each ethnic group to White (as a baseline group). The confounding factors age, parity and partner status were adjusted for in the models. Mode of delivery (vaginal versus caesarean) was also adjusted for in the analyses of perception of care. Prematurity was also adjusted for in the analyses relating to intrapartum and postnatal care. We did not adjust for language spoken at home as this is a key component of ethnic identity.

To minimise the non-response bias in the survey, the non-response weights were calculated based on the propensity modelling cell adjustment approach [30]. Women within the same National Health Service (NHS) trust (geographically based healthcare organisation) are likely to have similar experiences compared to women in different trusts which may lead to an overestimation of the standard errors within the clusters. To correct for this, survey data analysis commands were used to calculate robust standard errors which adjust the confidence intervals and p-values.

All of the logistic regressions were calculated using survey commands in Stata to account for the non-response weights and the clusters at NHS trust level. Categorical variables were summarized as frequencies and percentages.

Statistical analyses were carried out using STATA 11 software (StataCorp LP, College Station, TX).

## Results

A total of 24,319 women completed the survey, with a usable response rate of 52%. These were 84.8% White, 1.3% Mixed, 2.4% Indian, 2.5% Pakistani, 0.7% Bangladeshi, 0.7% Black Caribbean, 2.7% Black African, 5.0% Other ethnicity (excluding not known ethnicity) (Table 1). This is similar to the 2011 census data [2]. In a smaller parallel survey which also asked about country of origin, respondents of African origin reported coming from 29 different countries including Morocco, Angola, South Africa, Liberia and Somalia; and Other ethnicity included women from Afghanistan and the Philippines [31].

A comparison of the characteristics of women and their babies by ethnic group (Table 2) shows that compared to White women, those from minority ethnic groups tended to be younger, were more likely to be multiparous, without a partner, the language spoken at home was less likely to be English, and they were more likely to live with other family members. These differences were almost all highly statistically significant due, partly, to the sample size. Preterm birth and low birthweight were more common in minority ethnic groups, especially in babies born to Bangladeshi women.

**Table 2:** Characteristics of women and infants by ethnicity

**Characteristics**	**White**	**Mixed**	**Indian**	**Pakistani**	**Bangladeshi**	**Black Caribbean**	**Black African**	**Other ethnicity**	**Total**
	(n = 20633)	(n = 319)	(n = 584)	(n = 596)	(n = 163)	(n = 162)	(n = 659)	(n = 1203)	(24319)
**Age group**	*n (%)*	*n (%)*	*n (%)*	*n (%)*	*n (%)*	*n (%)*	*n (%)*	*n (%)*	*n (%)*
16-19	485 (2.4)	9 (2.8)	0 (0)	8 (1.3)	0 (0)	2 (1.2)	7 (1.1)	11 (0.9)	522 (2.1)
20-24	2575 (12.5)	44 (13.8)	34 (5.8)	84 (14.1)	31 (19.0)	27 (16.7)	49 (7.4)	114 (9.5)	2958 (12.2)
25-29	4699 (22.8)	74 (23.2)	161 (27.6)	233 (39.1)	57 (35.0)	40 (24.7)	145 (22.0)	312 (25.9)	5721 (23.5)
30-34	6727 (32.6)	100 (31.3)	266 (45.5)	177 (29.7)	47 (28.8)	44 (27.2)	254 (38.5)	386 (32.1)	8001 (32.9)
35-39	4718 (22.9)	65 (20.4)	103 (17.6)	82 (13.8)	26 (16.0)	33 (20.4)	154 (23.4)	282 (23.4)	5463 (22.5)
40+	1429 (6.9)	27 (8.5)	20 (3.4)*	12 (2.0)*	2 (1.2)*	16 (9.9)	50 (7.6)*	98 (8.1)*	1654 (6.8)
**Parity**									
Primiparous	10208 (49.9)	142 (45.5)	287 (50.0)	211 (35.8)	70 (43.2)	74 (46.3)	241 (37.3)	585 (49.6)	11818 (49.1)
Multiparous	10256 (50.1)	170 (54.5)	287 (50.0)	378 (64.2)*	92 (56.8)	86 (53.8)	405 (62.7)*	594 (50.4)	12268 (50.9)
**Partner**									
Yes	17935 (86.9)	227 (71.2)	486 (83.2)	454 (76.2)	128 (78.5)	74 (45.7)	433 (65.7)	959 (79.7)	20696 (85.1)
No	2698 (13.1)	92 (28.8)*	98 (16.8)*	142 (23.8)*	35 (21.5)*	88 (54.3)*	226 (34.3)*	244 (20.3)*	3623 (14.9)
Language spoken at home		244 (79.0)	244 (45.8)	203 (37.5)	46 (30.5)	152 (94.4)	288 (48.2)	539 (47.6)	21043 (88.4)
English	19327 (94.8)								
Other languages	1059 (5.2)	65 (21.0)*	289 (54.2)*	339 (62.5)*	105 (69.5)*	9 (5.6)	310 (51.8)*	594 (52.4)*	2770 (11.6)
**Living with family members other than a partner**									
Yes	918 (4.5)	27 (8.9)*	61 (10.9)*	91 (16.0)*	17 (11.0)*	21 (13.5)*	42 (6.7)*	87 (7.4)*	1264 (6.0)
**Gestation at birth**									
> = 37 weeks	19079 (93.0)	289 (91.7)	508 (88.2)	512 (88.1)	138 (85.7)	148 (91.9)	573 (89.1)	1064 (89.6)	22311 (92.4)
< 37 weeks	1444 (7.0)	26 (8.3)	68 (11.8)*	69 (11.9)*	23 (14.3)*	13 (8.1)	70 (10.9)*	123 (10.4)*	1836 (7.6)
**Birth weight**									
> = 2500 g	19543 (95.4)	295 (93.9)	512 (89.7)	507 (89.7)	134 (86.5)	149 (92.5)	561 (92.0)	1082 (93.2)	22783 (94.9)
<2500 g	936 (4.6)	19 (6.1)	59 (10.3)*	58 (10.3)*	21 (13.5)*	12 (7.5)	49 (8.0)*	79 (6.8)*	1233 (5.1)

*p<0.001.

### Antenatal care

The pattern of antenatal care received (Table 3) indicates less engagement with the health services in ethnic minority women. In particular, Black African, Black Caribbean and Pakistani women tended to start antenatal care later in pregnancy and have fewer antenatal checks and ultrasound scans. Pakistani women and those of Other ethnicity were also significantly less likely to attend antenatal classes.

**Table 3:** Access to antenatal services and care received during pregnancy by ethnicity

	**White**	**Mixed**	**Indian**	**Pakistani**	**Bangladeshi**	**Black Caribbean**	**Black African**	**Other Ethnicity**
**>12 weeks first saw HP**								
**n (%)**	859 (4.2)	18 (6.0)	33 (5.9)	**46 (8.3)**	8 (5.2)	**16 (10.2)**	**64 (10.5)**	**87 (7.6)**
aOR (95% CI)*	1	1.06 (0.64-1.75)	1.28 (0.88-1.86)	**1.82 (1.31-2.53)**	1.05 (0.50-2.19)	**1.89 (1.11-3.23)**	**2.19 (1.63-2.92)**	**1.80 (1.41-2.30)**
**MW first HP seen**								
**n (%)**	5128 (26.3)	**59 (19.8)**	**118 (21.7)**	171 (30.4)	**27 (17.2)**	**19 (12.5)**	**85 (13.7)**	**220 (19.4)**
aOR (95% CI)*	1	**0.66 (0.49-0.90)**	**0.76 (0.61-0.94)**	1.10 (0.91-1.34)	**0.54 (0.35-0.82)**	**0.44 (0.26-0.74)**	**0.44 (0.26-0.74)**	**0.68 (0.58-0.80)**
**>12 weeks booking appointment**								
**n (%)**	4375 (23.2)	**105 (36.6)**	141 (27.3)	**142 (27.7)**	33 (24.1)	**55 (37.4)**	**229 (40.2)**	**324 (31.1)**
aOR (95% CI)*	1	**1.73 (1.34-2.23)**	1.22 (0.99-1.49)	**1.30 (1.06-1.59)**	1.09 (0.73-1.62)	**1.66 (1.17-2.36)**	**2.02 (1.68-2.42)**	**1.46 (1.27-1.69)**
**Less than 10 AN check-ups**								
**n (%)**	14768 (77.6)	208 (73.8)	386 (74.5)	**425 (83.2)**	**118 (87.4)**	111 (77.1)	405 (77.4)	**851 (82.3)**
aOR (95% CI)*	1	0.86 (0.64-1.14)	0.84 (0.69-1.03)	**1.45 (1.14-1.85)**	**2.08 (1.22-3.54)**	1.08 (0.71-1.64)	1.03 (0.83-1.29)	**1.32 (1.11-1.57)**
**Dating scan (8–14 weeks)**								
**n (%)**	19506 (95.7)	288 (93.5)	**528 (93.5)**	**518 (92.7)**	147 (93.6)	149 (96.1)	**575 (91.7)**	**1054 (91.8)**
aOR (95% CI)*	1	0.68 (0.42-1.10)	**0.68 (0.48-0.96)**	**0.58 (0.42-0.82)**	0.73 (0.37-1.43)	1.31 (0.56-3.05)	**0.56 (0.41-0.78)**	**0.51 (0.40-0.64)**
**Down’s syndrome testing**								
**n (%)**	14731 (97.0)	223 (96.5)	415 (96.3)	**279 (95.2)**	**89 (96.7)**	**129 (97.7)**	**468 (95.7)**	877 (94.6)
aOR (95% CI)*	1	0.84 (0.40-1.78)	0.88 (0.52-1.48)	0.67 (0.38-1.18)	1.02 (0.32-3.29)	1.89 (0.57-6.28)	0.82 (0.51-1.31)	**0.58 (0.42-0.80)**
**Anomaly scan (20 weeks)**								
**n (%)**	20271 (99.3)	301 (98.0)	**553 (97.5)**	**551 (96.2)**	**148 (96.1)**	158 (100)	**597 (95.5)**	**1123 (97.1)**
aOR (95% CI)*	1	0.47 (0.20-1.10)	**0.29 (0.16-0.52)**	**0.21 (0.13-0.34)**	**0.24 (0.10-0.57)**	0	**0.18 (0.11-0.29)**	**0.26 (0.17-0.38)**
**2 or more scans during pregnancy**								
**n (%)**	20141 (98.6)	**302 (96.2)**	**548 (96.5)**	**543 (94.6)**	152 (96.2)	157 (98.1)	**587 (93.6)**	**1105 (95.3)**
aOR (95% CI)*	1	**0.40 (0.21-0.76)**	**0.37 (0.23-0.59)**	**0.27 (0.18-0.40)**	0.49 (0.21-1.15)	0.94 (0.27-3.29)	**0.22 (0.15-0.31)**	**0.30 (0.22-0.42)**
**Attended antenatal classes**								
**n (%)**	6371 (59.4)	93 (52.0)	198 (63.1)	**81 (25.6)**	31 (37.8)	46 (46.5)	177 (46.0)	**355 (52.0)**
aOR (95% CI)*	1	1.17 (0.76-1.81)	1.22 (0.88-1.69)	**0.34 (0.24-0.48)**	0.71 (0.37-1.38)	0.85 (0.49-1.47)	1.05 (0.77-1.43)	**0.77 (0.61-0.98)**

*Adjusted for woman’s age, parity and partner status HP health professional, MW midwife, AN antenatal. Figures in **bold** are statistically significant.

Women’s perceptions of antenatal care (Table 4) indicate that compared to White women, those from minority ethnic groups, particularly Pakistani women and those of Other ethnicity, were significantly less likely to report always being given the help they needed, spoken to in a way they could understand, and being sufficiently involved in decisions. They were also significantly less likely to report having a choice about place of birth and to rate their antenatal care as good overall.

**Table 4:** Perceptions of antenatal care by ethnicity

	**White**	**Mixed**	**Indian**	**Pakistani**	**Bangladeshi**	**Black Caribbean**	**Black African**	**Other ethnicity**
**Always given help needed by MW**								
**n (%)**	10852 (72.8)	165 (73.0)	294 (69.2)	**306 (66.4)**	**70 (61.4)**	72 (64.9)	305 (71.8)	**588 (67.8)**
aOR (95% CI)*	1	1.06 (0.77-1.47)	0.85 (0.68-1.06)	**0.79 (0.65-0.98)**	**0.61 (0.41-0.90)**	0.72 (0.48-1.09)	0.86 (0.69-1.08)	**0.81 (0.70-0.95)**
**Always spoken to in a way you could understand**								
**n (%)**	17216 (84.2)	249 (79.8)	471 (81.5)	**444 (76.4)**	122 (78.7)	127 (78.9)	522 (80.9)	**887 (75.9)**
aOR (95% CI)*	1	0.89 (0.65-1.21)	0.87 (0.70-1.10)	**0.71 (0.57-0.87)**	0.77 (0.51-1.15)	0.78 (0.52-1.17)	0.81 (0.65-1.01)	**0.59 (0.51-0.69)**
**Always involved enough in decisions**								
**n (%)**	15102 (74.7)	226 (73.9)	**398 (70.2)**	**363 (65.2)**	**84 (56.4)**	110 (70.1)	**438 (69.6)**	**782 (68.9)**
aOR (95% CI)*	1	1.09 (0.83-1.44)	**0.80 (0.66-0.97)**	**0.64 (0.53-0.77)**	**0.44 (0.31-0.63)**	0.82 (0.58-1.17)	**0.79 (0.66-0.96)**	**0.75 (0.65-0.86)**
**AN choice of place of birth**								
**n (%)**	15635 (84.8)	230 (80.1)	434 (81.6)	**356 (70.4)**	114 (78.1)	118 (81.9)	**444 (75.4)**	**896 (81.6)**
aOR (95% CI)*	1	0.90 (0.66-1.24)	0.87 (0.68-1.10)	**0.49 (0.40-0.61)**	0.72 (0.47-1.09)	0.97 (0.62-1.53)	**0.62 (0.51-0.77)**	**0.80 (0.68-0.95)**
**Good antenatal care overall rating**								
**n (%)**	18969 (92.7)	284 (89.3)	529 (91.5)	**515 (87.1)**	**143 (87.7)**	139 (86.9)	610 (93.7)	**1084 (91.2)**
aOR (95% CI)*	1	0.70 (0.48-1.02)	0.90 (0.65-1.23)	**0.54 (0.42-0.70)**	**0.59 (0.36-0.98)**	0.63 (0.39-1.01)	1.17 (0.83-1.64)	**0.80 (0.64-0.99)**

*Adjusted for woman’s age, parity, partner status and mode of delivery. Figures in **bold** are statistically significant.

### Labour and birth

Differences in care provided during labour and birth (Table 5) show that Black African, Pakistani and women of Other ethnicity were significantly less likely than White women to deliver at home or in a birth centre. Black African women and Asian women in general were significantly less likely to have pethidine, Pakistani women were significantly less likely to have an epidural. Nevertheless, there were no significant differences in women receiving the pain relief they wanted. An upright position for labour is thought to facilitate delivery [32], but this was less common in all minority ethnic groups especially Black Caribbean and Black African women. Black African women were significantly less likely to have a vaginal birth and were more likely to have an emergency caesarean section (22.6% compared with 13.9% in White women (data not shown)). Birth in water was uncommon overall, around five percent of women, but particularly so among the Asian groups at less than two percent.

**Table 5:** Care received during labour and birth by ethnicity

	**White**	**Mixed**	**Indian**	**Pakistani**	**Bangladeshi**	**Black Caribbean**	**Black African**	**Other ethnicity**
**Birth in a birth centre/at home vs hospital**								
** n (%)**	1348 (6.7)	11 (3.7)	26 (4.8)	**23 (4.2)**	10 (6.8)	7 (4.8)	**15 (2.7)**	**53 (4.7)**
aOR (95% CI)*	1	0.65 (0.34-1.26)	0.71 (0.47-1.09)	**0.64 (0.41-0.99)**	1.03 (0.52-2.04)	0.78 (0.36-1.71)	**0.41 (0.25-0.70)**	**0.68 (0.51-0.92)**
**Pethidine or similar for pain relief**								
** n (%)**	5530 (30.7)	77 (28.7)	**125 (25.4)**	**119 (23.2)**	**34 (23.6)**	41 (28.7)	**129 (24.6)**	**279 (26.5)**
aOR (95% CI)*	1	0.85 (0.64-1.13)	**0.75 (0.60-0.93)**	**0.67 (0.53-0.83)**	**0.64 (0.42-0.95)**	0.77 (0.52-1.15)	**0.77 (0.62-0.96)**	**0.81 (0.69-0.94)**
**Epidural or similar for pain relief**								
** n (%)**	5294 (29.4)	77 (28.7)	147 (29.8)	**101 (19.6)**	28 (19.4)	35 (24.5)	158 (30.1)	**342 (32.5)**
aOR (95% CI)*	1	0.94 (0.70-1.28)	1.01 (0.82-1.24)	**0.70 (0.56-0.89)**	0.65 (0.42-1.01)	0.78 (0.52-1.17)	1.23 (0.99-1.52)	**1.17 (1.01-1.35)**
**Vaginal delivery**								
** n (%)**	15520 (75.7)	228 (72.6)	418 (73.3)	441 (77.1)	125 (79.1)	121 (75.2)	**404 (64.6)**	856 (73.0)
aOR (95% CI)*	1	0.86 (0.66-1.12)	0.93 (0.77-1.13)	0.98 (0.79-1.20)	1.08 (0.72-1.63)	0.95 (0.64-1.40)	**0.58 (0.48-0.69)**	0.94 (0.82-1.09)
**Sitting/standing/on side vs lying at birth**								
** n (%)**	4949 (32.9)	64 (28.7)	**114 (27.4)**	**130 (29.5)**	**34 (27.4)**	**24 (20.5)**	**85 (21.3)**	**244 (29.2)**
aOR (95% CI)*	1	0.88 (0.65-1.18)	**0.72 (0.58-0.90)**	**0.68 (0.55-0.85)**	**0.67 (0.45-0.99)**	**0.60 (0.40-0.91)**	**0.53 (0.42-0.67)**	**0.81 (0.70-0.95)**
**Birth in water**								
** n (%)**	809 (5.2)	10 (4.3)	**8 (1.9)**	**1 (0.2)**	**2 (1.6)**	9 (7.4)	**9 (2.2)**	**27 (3.1)**
aOR (95% CI)*	1	1.13 (0.56-2.29)	**0.41 (0.20-0.84)**	**0.05 (0.01-0.36)**	**0.16 (0.04-0.66)**	1.74 (0.86-3.50)	**0.40 (0.20-0.79)**	**0.53 (0.35-0.81)**
**Birth in water/floor vs in bed**								
** n (%)**	1550 (10.2)	16 (7.0)	**18 (4.3)**	**15 (3.4)**	**5 (4.0)**	15 (12.5)	**15 (3.6)**	**55 (6.4)**
aOR (95% CI)*	1	0.90 (0.51-1.57)	**0.43 (0.27-0.70)**	**0.29 (0.17-0.50)**	**0.34 (0.13-0.91)**	1.47 (0.84-2.59)	**0.35 (0.20-0.59)**	**0.60 (0.45-0.81)**

*Adjusted for woman’s age, parity, partner status and prematurity. Figures in bold are statistically significant.

Women’s perceptions of intrapartum care (Table 6) show that the majority felt able to move around during labour but this was significantly less so in Bangladeshi, Pakistani and Black African women and those of Other ethnicity. Similarly, while confidence and trust in staff was generally very high and most women felt that their partner was made welcome, these were significantly less evident among Bangladeshi and Pakistani women. One-to-one care in labour is considered optimal [33], however, in early labour this is often not available. Being left alone in labour and shortly after the birth at a time when it worried them was significantly more common in Asian, Black African and women of Other ethnicity. Similarly, although almost all women felt that they were always spoken to in a way they could understand and sufficiently involved in decisions, communication was poorer for all minority ethnic groups, especially Pakistani, Bangladeshi and Black Caribbean women. Overall, minority ethnic group women were significantly less likely than White women to rate care in labour and birth as good.

**Table 6:** Women’s perceptions of labour and birth care by ethnicity

	**White**	**Mixed**	**Indian**	**Pakistani**	**Bangladeshi**	**Black Caribbean**	**Black African**	**Other ethnicity**
**Had met staff before**								
** n (%)**	4478 (22.0)	**91 (29.7)**	**183 (32.8)**	**189 (33.5)**	**57 (38.3)**	**51 (31.9)**	**214 (34.2)**	**473 (41.3)**
aOR (95% CI)*	1	**1.61 (1.23-2.10)**	**1.72 (1.43-2.07)**	**1.63 (1.35-1.97)**	**2.09 (1.48-2.96)**	**1.52 (1.06-2.17)**	**1.70 (1.42-2.03)**	**2.45 (2.15-2.79)**
**Able to move around/choose position most of the time**								
** n (%)**	14319 (91.4)	215 (89.6)	398 (90.9)	**407 (88.9)**	**102 (81.6)**	100 (84.0)	**388 (87.8)**	**816 (88.1)**
aOR (95% CI)*	1	0.97 (0.74-1.27)	0.86 (0.71-1.04)	**0.67 (0.56-0.81)**	**0.52 (0.36-0.74)**	0.85 (0.60-1.20)	**0.69 (0.57-0.83)**	**0.82 (0.72-0.94)**
**Received pain relief you wanted to at least some extent**								
** n (%)**	14435 (84.3)	202 (84.9)	379 (82.2)	379 (82.4)	103 (81.7)	105 (82.0)	383 (81.0)	799 (82.5)
aOR (95% CI)*	1	1.22 (0.82-1.80)	0.91 (0.71-1.18)	0.89 (0.69-1.15)	0.85 (0.53-1.36)	0.82 (0.51-1.32)	0.85 (0.66-1.08)	0.92 (0.76-1.11)
**Confidence and trust in staff**								
** n (%)**	19701 (96.4)	303 (95.9)	547 (95.1)	**549 (93.8)**	**145 (92.4)**	149 (93.1)	609 (95.5)	**1118 (95.2)**
aOR (95% CI)*	1	0.97 (0.55-1.72)	0.71 (0.48-1.06)	**0.61 (0.43-0.88)**	**0.50 (0.27-0.92)**	0.53 (0.28-1.00)	0.81 (0.54-1.20)	**0.72 (0.54-0.97)**
**Partner made welcome**								
** n (%)**	19806 (98.0)	296 (96.1)	549 (97.3)	**541 (95.9)**	**149 (93.7)**	143 (96.6)	569 (96.8)	1122 (98.2)
aOR (95% CI)*	1	0.62 (0.34-1.15)	0.67 (0.40-1.14)	**0.60 (0.39-0.94)**	**0.34 (0.18-0.67)**	0.69 (0.27-1.79)	0.74 (0.44-1.24)	1.10 (0.68-1.78)
**Left alone and worried in labour**								
** n (%)**	2288 (12.3)	42 (15.3)	71 (14.7)	**108 (24.5)**	**26 (20.8)**	23 (16.3)	**86 (16.8)**	**195 (20.4)**
aOR (95% CI)*	1	1.38 (0.97-1.98)	1.24 (0.95-1.61)	**2.28 (1.80-2.87)**	**1.72 (1.09-2.73)**	1.21 (0.76-1.92)	**1.34 (1.04-1.73)**	**1.83 (1.53-2.18)**
**Left alone and worried after birth**								
** n (%)**	1001 (5.8)	22 (8.7)	**48 (10.4)**	**81 (19.6)**	**13 (11.6)**	12 (9.2)	**64 (13.1)**	**124 (14.0)**
aOR (95% CI)*	1	1.39 (0.87-2.23)	**1.92 (1.40-2.63)**	**3.49 (2.67-4.57)**	**2.12 (1.17-3.85)**	1.58 (0.85-2.93)	**2.47 (1.85-3.30)**	**2.73 (2.21-3.38)**
**Spoken to in a way could understand**								
** n (%)**	19980 (97.8)	302 (95.9)	559 (97.7)	**558 (96.2)**	**146 (94.8)**	153 (95.0)	615 (96.4)	1151 (97.6)
aOR (95% CI)*	1	0.54 (0.28-1.01)	0.95 (0.54-1.67)	**0.60 (0.38-0.94)**	**0.46 (0.22-0.97)**	0.57 (0.27-1.17)	0.64 (0.41-1.00)	1.03 (0.68-1.58)
**Involved enough in decisions**								
** n (%)**	18991 (94.3)	287 (94.4)	531 (94.8)	**501 (90.4)**	135 (90.6)	**139 (88.5)**	583 (94.0)	1069 (93.0)
aOR (95% CI)*	1	1.04 (0.62-1.73)	1.10 (0.74-1.63)	**0.58 (0.43-0.78)**	0.62 (0.35-1.10)	**0.50 (0.30-0.83)**	1.07 (0.74-1.53)	0.83 (0.65-1.07)
**Labour and birth overall rating good**								
** n (%)**	19152 (94.0)	**280 (90.6)**	**514 (89.7)**	**497 (84.8)**	**133 (83.6)**	**142 (88.2)**	590 (92.2)	**1080 (90.9)**
aOR (95% CI)*	1	**0.65 (0.44-0.98)**	**0.54 (0.41-0.72)**	**0.38 (0.30-0.48)**	**0.35 (0.22-0.54)**	**0.51 (0.31-0.84)**	0.81 (0.59-1.11)	**0.64 (0.51-0.79)**

*Adjusted for woman’s age, parity, partner status, mode of delivery and prematurity. Figures in **bold** are statistically significant.

### Postnatal care

Differences in postnatal care (Table 7) indicate that all minority ethnic groups were more likely to experience longer stays in hospital compared to White women, especially Asian and Black African women. However, they were more likely to be unhappy about their length of stay, Pakistani and Indian women significantly so, feeling that their length of stay was too long.

**Table 7:** Postnatal care received by ethnicity

	**White**	**Mixed**	**Indian**	**Pakistani**	**Bangladeshi**	**Black Caribbean**	**Black African**	**Other ethnicity**
**PN stay 3 days or more**								
** n (%)**	5658 (28.5)	102 (32.8)	**206 (36.6)**	**190 (33.8)**	51 (32.5)	50 (32.1)	**242 (38.5)**	**381 (33.1)**
aOR (95% CI)*	1	1.15 (0.87-1.51)	**1.41 (1.17-1.70)**	**1.47 (1.21-1.79)**	1.27 (0.88-1.83)	1.13 (0.78-1.65)	**1.63 (1.36-1.96)**	**1.16 (1.01-1.34)**
**Felt length of stay too long/short vs about right**								
** n (%)**	5342 (27.4)	76 (25.3)	**177 (32.7)**	**189 (34.9)**	42 (29.0)	48 (32.0)	170 (28.6)	311 (28.7)
aOR (95% CI)*	1	0.85 (0.64-1.12)	**1.28 (1.06-1.55)**	**1.35 (1.12-1.63)**	1.13 (0.78-1.63)	1.22 (0.85-1.74)	1.08 (0.89-1.31)	1.06 (0.92-1.23)
**Baby put to breast at least once**								
** n (%)**	16763 (81.8)	**290 (92.1)**	**563 (97.9)**	**538 (91.2)**	**156 (98.1)**	**150 (94.3)**	**611 (95.2)**	**1144 (96.8)**
aOR (95% CI)*	1	**3.32 (2.12-5.18)**	**12.65 (7.01-22.84)**	**3.64 (2.67-4.95)**	**17.49 (5.40-56.62)**	**7.90 (3.69-16.88)**	**6.75 (4.48-10.16)**	**8.13 (5.67-11.67)**
**Baby was fed by any breast milk vs only formula milk in the first few days**								
** n (%)**	15667 (76.7)	**279 (88.9)**	**530 (92.2)**	**475 (80.5)**	**141 (88.7)**	**144 (90.6)**	**592 (92.5)**	**1071 (90.8)**
aOR (95% CI)*	1	**3.14 (2.11-4.68)**	**4.12 (2.96-5.71)**	**1.93 (1.53-2.43)**	**3.23 (1.88-5.55)**	**5.19 (2.89-9.33)**	**5.27 (3.78-7.36)**	**3.45 (2.75-4.33)**
**Generally had consistent advice from midwives and others**								
** n (%)**	15230 (77.9)	248 (82.4)	**472 (83.0)**	479 (83.4)	120 (77.9)	132 (85.2)	**563 (91.0)**	**998 (86.3)**
aOR (95% CI)*	1	1.21 (0.88-1.67)	**1.37 (1.09-1.72)**	1.22 (0.97-1.54)	0.84 (0.56-1.25)	1.54 (0.97-2.42)	**2.63 (1.96-3.52)**	**1.82 (1.51-2.18)**
**Given breast feeding support**								
** n (%)**	16983 (85.9)	258 (85.1)	478 (84.3)	482 (84.9)	130 (83.3)	128 (83.7)	**561 (90.5)**	**1029 (88.8)**
aOR (95% CI)*	1	0.90 (0.64-1.26)	0.87 (0.68-1.10)	0.94 (0.73-1.20)	0.80 (0.51-1.24)	0.88 (0.56-1.38)	**1.55 (1.17-2.07)**	**1.28 (1.05-1.56)**
**Feeding advice received**								
** n (%)**	15421 (87.4)	232 (84.1)	**449 (83.8)**	**423 (80.9)**	117 (81.3)	122 (83.6)	496 (85.7)	933 (87.2)
aOR (95% CI)*	1	0.75 (0.53-1.07)	**0.76 (0.60-0.98)**	**0.70 (0.55-0.89)**	0.68 (0.44-1.05)	0.90 (0.57-1.42)	0.93 (0.73-1.20)	0.95 (0.78-1.15)
**Information on recovery after birth**								
** n (%)**	16218 (82.0)	247 (80.5)	462 (83.4)	**450 (79.9)**	126 (81.3)	124 (80.5)	**548 (87.5)**	**967 (85.4)**
aOR (95% CI)*	1	0.88 (0.65-1.18)	1.07 (0.85-1.36)	**0.80 (0.64-0.99)**	0.87 (0.57-1.32)	0.92 (0.61-1.40)	**1.41 (1.09-1.82)**	**1.29 (1.08-1.55)**
**> = 5 MW visits**								
** n (%)**	4961 (24.8)	60 (20.1)	124 (22.3)	**111 (19.9)**	**22 (13.8)**	31 (19.5)	**115 (18.5)**	259 (22.8)
aOR (95% CI)*	1	0.76 (0.57-1.03)	0.84 (0.69-1.04)	**0.80 (0.64-0.99)**	**0.46 (0.29-0.73)**	0.70 (0.47-1.06)	**0.64 (0.52-0.80)**	0.89 (0.77-1.04)
**Given information/explanations needed**								
** n (%)**	10403 (52.4)	172 (55.5)	299 (52.8)	297 (52.8)	75 (48.4)	88 (56.4)	**393 (62.2)**	637 (55.2)
aOR (95% CI)*	1	1.10 (0.86-1.40)	1.02 (0.86-1.21)	0.91 (0.76-1.09)	0.77 (0.55-1.08)	1.07 (0.77-1.50)	**1.38 (1.16-1.65)**	1.09 (0.96-1.24)
**Had postnatal check**								
** n (%)**	18096 (89.2)	279 (89.1)	499 (88.6)	**469 (79.8)**	**122 (77.2)**	**127 (81.9)**	566 (88.4)	1030 (87.8)
aOR (95% CI)*	1	1.09 (0.73-1.61)	0.95 (0.72-1.24)	**0.53 (0.42-0.66)**	**0.44 (0.30-0.66)**	**0.62 (0.40-0.94)**	0.98 (0.76-1.26)	0.87 (0.72-1.06)

*Adjusted for woman’s age, parity, partner status and prematurity. Figures in **bold** are statistically significant.

Breastfeeding was more common in all minority ethnic groups compared to White women. Initiation of breastfeeding was 98% in Bangladeshi and Indian women compared to 92% in White women. Over the first few days the disparity in rates of breastfeeding declined but was still quite marked. Women did not differ in their perceptions of breastfeeding support, consistent advice or information about recovery after birth, except that Black African women and those of Other ethnicity rated it more highly. However, Indian and Pakistani women were significantly more likely to report not receiving enough feeding advice.

Bangladeshi, Black African and Pakistani women were significantly less likely to have had five or more home visits by their midwife after hospital discharge. Postnatal checks of their own health in the weeks after birth were also less likely for Bangladeshi, Pakistani and Black Caribbean women.

A comparison of perceptions of postnatal care (Table 8) shows that all groups of Asian women and those of Other ethnicity were significantly less likely to feel that they were always treated with kindness in hospital and after discharge all minority ethnic groups were significantly less likely than White women to see a midwife as much as they wanted. Many Pakistani and Bangladeshi women reported not having enough advice about emotional changes associated with childbirth. Overall, ratings of postnatal care were not as positive as for antenatal or intrapartum care, and were significantly poorer among Bangladeshi, Pakistani, Indian, Black Caribbean and women of Other ethnicity.

**Table 8:** Women’s perceptions of postnatal care by ethnicity

	**White**	**Mixed**	**Indian**	**Pakistani**	**Bangladeshi**	**Black Caribbean**	**Black African**	**Other ethnicity**
**In hospital always treated with kindness**								
** n (%)**	12616 (63.6)	203 (65.9)	**325 (57.9)**	345 (60.8)	**75 (48.4)**	94 (60.3)	397 (63.0)	**698 (61.0)**
aOR (95% CI)*	1	1.12 (0.87-1.45)	**0.81 (0.68-0.97)**	0.86 (0.72-1.04)	**0.50 (0.35-0.70)**	0.84 (0.60-1.17)	0.92 (0.75-1.07)	**0.87 (0.76-0.99)**
**After hospital discharge, help given when contacted MW**								
** n (%)**	13282 (96.2)	216 (94.3)	390 (94.9)	405 (95.3)	109 (96.5)	102 (92.7)	416 (96.5)	**822 (94.6)**
aOR (95% CI)*	1	0.60 (0.32-1.13)	0.80 (0.50-1.28)	0.83 (0.51-1.33)	0.96 (0.35-2.65)	0.54 (0.26-1.13)	1.10 (0.64-1.89)	**0.72 (0.52-0.99)**
**Saw MW as much as wanted**								
** n (%)**	15732 (78.1)	**213 (69.8)**	**316 (56.6)**	**297 (52.4)**	**78 (50.6)**	**96 (60.8)**	**320 (52.1)**	**613 (53.0)**
aOR (95% CI)*	1	**0.67 (0.52-0.88)**	**0.36 (0.30-0.43)**	**0.32 (0.27-0.38)**	**0.28 (0.20-0.39)**	**0.51 (0.36-0.71)**	**0.32 (0.27-0.37)**	**0.31 (0.27-0.35)**
**Enough information on emotional changes**								
** n (%)**	15279 (80.1)	226 (77.4)	413 (77.1)	**377 (69.7)**	**99 (67.3)**	117 (76.5)	462 (77.6)	851 (78.1)
aOR (95% CI)*	1	0.81 (0.60-1.08)	0.83 (0.67-1.03)	**0.58 (0.48-0.71)**	**0.53 (0.37-0.76)**	0.88 (0.59-1.29)	0.85 (0.69-1.05)	0.86 (0.73-1.00)
**Postnatal overall rating good**								
** n (%)**	18024 (88.7)	274 (86.7)	**481 (84.5)**	**485 (82.6)**	**122 (76.7)**	**126 (79.7)**	579 (89.8)	**1010 (86.3)**
aOR (95% CI)*	1	0.79 (0.56-1.13)	**0.67 (0.53-0.85)**	**0.61 (0.48-0.76)**	**0.42 (0.29-0.62)**	**0.54 (0.36-0.80)**	1.06 (0.81-1.39)	**0.75 (0.63-0.90)**

*Adjusted for woman’s age, parity, partner status, mode of delivery and prematurity. Figures in **bold** are statistically significant.

## Discussion

This study has shown that women in all minority ethnic groups report a poorer experience of maternity care than White women. This is apparent in the antenatal, intrapartum and postnatal stages of care and is consistent with two similar large studies, one using the Healthcare Commission (HCC) survey data from 2007 [15] and one using the Millennium Cohort Study of babies born in 2000–2001 [34]. These studies also reported higher rates of late booking, fewer antenatal checks and ultrasound scans, and less choice regarding place of birth among minority ethnic group women. The 2007 survey found that during labour minority ethnic group women had less confidence and trust in staff, were more likely to be left alone and worried, and Asian and Black women were more likely to have an unplanned caesarean section. Postnatally, minority ethnic group women had higher breastfeeding rates, longer lengths of stay, and were less likely to see a midwife at home as much as they wished [15]. Other studies have found similar results across a range of factors [13,14,16,35].

Direct comparison of these more recent data with the 2007 survey is difficult due to differences in question design and the different grouping of ethnicity. Nevertheless, it appears that very little has changed, although with this more recent study we can better appreciate differences between Black African and Black Caribbean women and between Indian, Pakistani and Bangladeshi women. The direction and size of the disparity between women from ethnic minorities and White women is very similar to that reported in earlier studies.

The attitudes of staff and the nature of interaction are a critical aspect of care for all women [36]. However, the experience of women from minority ethnic groups was more likely to be negative in relation to communication and decision-making, compared to that of White women. An Australian study examining the role of culture and communication in Vietnamese, Turkish and Filipino women giving birth found that they were more concerned about care being rushed, unkind and unsupportive and less about caregivers being unfamiliar with their cultural practices [37]. Unhelpful attitudes of health professionals can lead to women being less willing to access care, attending later for antenatal care and later when in labour [38].

Limitations of this study include the 52% response to the survey and that women who were less familiar with English would have been less likely to complete it. However, 3686 women from minority ethnic groups responded and the differential non-response was corrected for in the analysis. We had no information about duration of residence in the UK or about country of origin. A further limitation is the lack of any variable relating to SES. However, studies have shown that, although SES is associated with ethnicity, it does not explain differences in mortality [8,9]. Analysis of similar population based data showed that even with adjustment for factors including Index of Multiple Deprivation (IMD) (a measure reflecting disadvantaged area status) minority ethnic women were more critical about some aspects of their care [39]. However, it is not clear to what extent SES may explain differences in perceptions and process of care within different ethnic groups studied.

The strengths of this study are its size and more detailed breakdown by ethnic group. There were noticeable and significant differences between the different Asian groups, and between the two Black groups. For example, Pakistani women were significantly more likely to have poor experience of antenatal care, and Bangladeshi women of postnatal care, whereas Indian women were much more similar to White women in these respects. Similarly, Black African women were more likely to book late, have fewer ultrasound scans, an unplanned caesarean, be left alone and worried in labour and after the birth compared to Black Caribbean women. These differences may relate to recency of migration, availability of family and social support, differences in religious beliefs, cultural attitudes and expectations, and the extent of societal integration more broadly. It was interesting that women of Mixed ethnicity were not significantly different from White women in most of the outcomes investigated. The only exceptions to this were late booking, a less positive overall rating of labour and birth care and not seeing a midwife as much as wanted after hospital discharge.

Postnatal differences tended to be in the other direction. For example, a length of stay of three days or more was more common in Asian and Black African women, possibly reflecting an increased need for care, as with the higher proportion of Black African women having a caesarean section. It may also reflect the expectation among Asian groups that the immediate postnatal period is a time for rest [40] although Indian and Pakistani women were more likely to feel that their length of stay was too long. Similarly, initiation of breastfeeding ('baby put to breast at least once’) was more common among minority ethnic group women than White women, reflecting the cultural norms in those groups [41]. Black African women and those from the Other ethnic group were more likely than White women to consider that they had received breastfeeding support and information regarding their own recovery.

The greatest differences between minority ethnic group women and White women related to timing of the first contact with health professionals and booking for maternity care and being left alone in labour or after birth at a time when it worried them. Factors contributing to these differences have been explored elsewhere [13,16,42,43]. They include discrimination and stereotyping by health professionals and difficulties in understanding and culture on both sides. From the health professionals point of view, it can be stressful, time-consuming and frustrating caring for women who do not speak English and who may have different cultural expectations [42].

Implications for care are principally for health professionals to recognise the diversity within groups. For example, Asian and Black women vary in their cultural and religious beliefs, their economic and migration histories. This is reflected in the way care is accessed, preferences for traditional rather than medical models and the extent to which Western values are adopted. There may be too much of an expectation by health professionals that women and their families will adapt to the dominant culture, rather than the maternity service being responsive and sensitive to the needs of a multicultural population. It is disappointing that ethnic minority women’s experience of maternity care does not appear to have improved appreciably over the last decade, particularly as an increasing proportion of women using the maternity services were born outside the UK [1].

## Conclusions

Women in all minority ethnic groups had a poorer experience of maternity services than White women. That this was still the case following publication of a number of national policy documents and local initiatives to improve childbirth experiences and outcomes for ethnic minority women is a cause for concern. Previous research has demonstrated the importance of individualised care and the potential of advocates and link workers in improving the experience of care of minority ethnic groups [12]. Further survey work is planned to examine the moderating effects of recency of migration and the experiences of white women who were not born in the UK. Further qualitative research may be helpful in illuminating the experiences of women who do not speak English, and the moderating effect of social support and integration.

## Abbreviations

SES: Socioeconomic status; NHS: National Health Service; HCC: Health care commission; CQC: Care quality commission; IMD: Index of multiple deprivation; aOR: Adjusted odds ratio; HP: Health professional; MW: Midwife; AN: Antenatal; PN: Postnatal.

## Competing interests

The authors declare that they have no competing interests.

## Authors’ contributions

JH conducted some of the analyses and drafted the manuscript. HG carried out the majority of the statistical analyses. MR oversaw the design, management and coordination of the study and helped to draft the manuscript. All authors read and approved the final manuscript.

## Pre-publication history

The pre-publication history for this paper can be accessed here:

http://www.biomedcentral.com/1471-2393/13/196/prepub
